# On the Approximation of Generalized Lipschitz Function by Euler Means of Conjugate Series of Fourier Series

**DOI:** 10.1155/2013/508026

**Published:** 2013-11-26

**Authors:** Jitendra Kumar Kushwaha

**Affiliations:** Department of Pure & Applied Mathematics, Guru Ghasidas University, Koni, Bilaspur 495009, India

## Abstract

Approximation theory is a very important field which has various applications in pure and applied mathematics. The present study deals with a new theorem on the approximation of functions of Lipschitz class by using Euler's mean of conjugate series of Fourier series. In this paper, the degree of approximation by using Euler's means of conjugate of functions belonging to Lip (*ξ*(*t*), *p*) class has been obtained. Lip*α* and Lip (*α*, *p*) classes are the particular cases of Lip (*ξ*(*t*), *p*) class. The main result of this paper generalizes some well-known results in this direction.

## 1. Introduction and Definitions

Let  *f*  be periodic with period  2*π*  and integrable in the sense of Lebesgue. The Fourier series associated with  *f*  at the point  *x*  is given by

(1) $$\begin{array}{c} f\left(x\right)\sim \frac{1}{2}a_{0}+\overset{\infty }{\underset{n=1}{\sum }}\left(a_{n}\cos nx+b_{n}\sin nx\right)=\overset{\infty }{\underset{n=0}{\sum }}A_{n}\left(x\right) \end{array}$$

with partial sums  *s*_*n*_(*f*; *x*). The conjugate series of (1) is given by

(2) $$\begin{array}{c} B_{n}\left(x\right)=\overset{\infty }{\underset{n=1}{\sum }}\left(a_{n}\sin nx-b_{n}\cos nx\right) \end{array}$$

with partial sums$\;{\overset{\sim }{s}}_{n}(f;x)$. Throughout this paper, we call (2) as conjugate series of Fourier series of function  *f*. If  *f*  is Lebesgue integrable, then

(3) f~(x)=−12π∫0πψ(t)cot⁡(t2)dt=−12πlim⁡ε→0∫επψ(t)cot⁡(t2)dt

exists for almost all  *x*  (Hardy [1], page 131).$\;\overset{\sim }{f}(x)\;$is called the conjugate function of  *f*(*x*).

A function  *f* ∈ Lip *α*  if

(4) $$\begin{array}{c} \left|f\left(x+t\right)-f\left(x\right)\right|=O\left({\left|t\right|}^{\alpha }\right)\;\text{for}\;0<\alpha \leq 1. \end{array}$$

*f* ∈ Lip (*α*, *p*),  *p* > 1  consider that if

(5) {∫02π|f(x+t)−f(x)|pdx}1/p=O(|t|α),           0<α≤1,  p≥1  

(Definition  5.38  of Chandra [2]).

Given a positive increasing function  *ξ*(*t*),  *f* ∈ Lip (*ξ*(*t*), *p*),

(6) (∫02π|(f(x+t)−f(x))|pdx)1/p≤M(  ξ(t)t−1/p),     p>1,

where  *M*  is a positive number independent of  *x*  and  *t*.

In case  *ξ*(*t*) = *t* ^*α*^, then Lip (*ξ*(*t*), *p*)  coincides with Lip (*α*, *p*). If  *p* → *∞*  in Lip (*α*, *p*), then it coincides with Lip *α*.

*L*_*∞*_-norm of a function  *f* : *R* → *R*  is defined by

(7) $$\begin{array}{c} {||f||}_{\infty }=\sup\left\{\left|f\left(x\right):x\in R\right|\right\}. \end{array}$$

*L*_*p*_-norm is defined by

(8) $$\begin{array}{c} {||f||}_{p}={\left(\overset{2\pi }{\underset{0}{\int }}{\left|f\left(x\right)\right|}^{p}dx\right)}^{1/p},\;p\geq 1. \end{array}$$

The degree of approximation of a function  *f* : *R* → *R*  by a trigonometric polynomial  *t*_*n*_  of order  *n*  under sup norm || ||_*∞*_ is defined by ([1], page 114-115])

(9) $$\begin{array}{c} {||t_{n}-f||}_{\infty }=\sup\left\{\left|t_{n}\left(x\right)-f\left(x\right)\right|:x\in R\right\}, \end{array}$$

and  *E*_*n*_(*f*)  of a function  *f* ∈ *L*_*p*_  is given by

(10) $$\begin{array}{c} E_{n}\left(f\right)=\min{||t_{n}-f||}_{p}. \end{array}$$

Let  {*S*_*n*_}  be the sequence of partial sums of the given series  ∑_*n*=0_^*∞*^ *u*_*n*_. Then, for  *q* > 0, the Euler (*E*, *q*) means of  {*S*_*n*_}  are defined to be

(11) $$\begin{array}{c} W_{n}={\left(1+q\right)}^{-n}\overset{n}{\underset{k=0}{\sum }}\left(\begin{array}{c} n\\ k \end{array}\right)q^{n-k}S_{k}. \end{array}$$

The series is said to be Euler (*E*, *q*) summable to  *S*  provided that the sequence  {*W*_*n*_}  converges to  *S*  as  *n* → +*∞*.

We write

(12) $$\begin{array}{c} \psi \left(t\right)=f\left(x+t\right)-f\left(x-t\right),\\ {\overset{\sim }{\sigma }}_{n}\left(f;x\right)={\left(1+q\right)}^{-n}\overset{n}{\underset{k=0}{\sum }}\left(\begin{array}{c} n\\ k \end{array}\right)q^{n-k}{\overset{\sim }{S}}_{k},\\ \overset{\sim }{S}\left(t\right)=\overset{n}{\underset{k=0}{\sum }}\left(\begin{array}{c} n\\ k \end{array}\right)q^{n-k}\cos\left(k+\frac{1}{2}\right)t,\\ R\left(t\right)=\sin\left\{\frac{t}{2}+n\text{ta}{\text{n}}^{-1}\left(\frac{\sin t}{q+\cos t}\right)\right\}. \end{array}$$

## 2. Main Theorem

Hardy [1] established a theorem on (*C*, *α*), (*α* > 0) summability of the series. Harmonic summability is weaker than (*C*, *α*) summability. Iyengar [3] proved a theorem on harmonic summability of a Fourier series. The result of Iyengar [3] has been generalized by several researchers like Siddiqi [4], Pati [5], Lal and Kushwaha [6], and Rajagopal [7], for Nörlund means.

Alexits [8] proved the following theorem concerning the degree of approximation of a function  *f* ∈ Lip *α*  by the (*C*, *δ*) means of its Fourier series.

**Theorem 2 A**

If a periodic function  *f* ∈ Lip *α*,  0 < *α* ≤ 1, then the degree of approximation of the (*C*, *δ*) means of its Fourier series for  0 < *α* < *δ* ≤ 1  is given by

(13) $$\begin{array}{c} \underset{0\leq x\leq 2\pi }{\max}\left|f\left(x\right)-\sigma_{n}^{\delta }\left(x\right)\right|=O\left(\frac{1}{n^{\alpha }}\right) \end{array}$$

and for  0 < *α* ≤ *δ* ≤ 1  is given by

(14) $$\begin{array}{c} \underset{0\leq x\leq 2\pi }{\max}\left|f\left(x\right)-\sigma_{n}^{\delta }\left(x\right)\right|=O\left(\frac{\log n}{n^{\alpha }}\right), \end{array}$$

where  *σ*_*n*_^*δ*^(*x*)  are the (*C*, *δ*) means of the partial sums of (2).

Later on, Hölland et al. [9] extended Theorem A to functions belonging to  *C*∗[0,2*π*], the class of  2*π*-periodic continuous functions on [0,2*π*], using Nörlund means of Fourier series. Their theorem is as follows.

**Theorem 2 B**

If  *w*(*t*)  is the modulus of continuity of  *f* ∈ *C*∗[0,2*π*], then the degree of approximation of  *f*  by the Nörlund means of the Fourier series for f is given by

(15) $$\begin{array}{c} E_{n}=\underset{0\leq t\leq 2\pi }{\max}\left|f\left(t\right)-T_{n}\left(t\right)\right|=O\left(\frac{1}{p_{n}}\overset{n}{\underset{k=1}{\sum }}\frac{p_{k}w\left(1/k\right)}{k}\right), \end{array}$$

where  *T*_*n*_  are the  (*N*, *p*_*n*_)  means of Fourier series of  *f*.

Hölland et al. [9] have shown that Theorem B reduces to Theorem A if we deal with Cesàro means of order  *δ*  and consider a function  *f* ∈ Lip *α*,  0 < *α* ≤ 1. Working in same direction we prove the following theorem.

**Theorem 1**

If  *f* : *R* → *R*  is a  2*π*  periodic, Lebesgue integrable and belonging to Lip (*ξ*(*t*), *p*)  for > 1  and if

(16) $$\begin{array}{c} {\left\{\overset{1/\sqrt{n}}{\underset{0}{\int }}{\left(\frac{t\left|\xi \left(t\right)\right|}{t^{1/p}}\right)}^{p}dt\right\}}^{1/p}=O\left(\xi \left(\frac{1}{\sqrt{n}}\right)\right), \end{array}$$

(17) $$\begin{array}{c} {\left\{\overset{\pi }{\underset{1/\sqrt{n}}{\int }}{\left(\frac{\left|\xi \left(t\right)\right|}{t^{1/p+2}}\right)}^{p}dt\right\}}^{1/p}=O\left(\xi \left(\frac{1}{\sqrt{n}}\right)n\right) \end{array}$$

conditions (16) and (17) hold uniformly in  *x*, then degree of approximation of$\;\overset{\sim }{f}\left(x\right)$, conjugate of  *f* ∈ Lip {*ξ*(*t*), *p*}, by Euler (*E*, *q*) mean

(18) $$\begin{array}{c} {\overset{\sim }{\sigma }}_{n}\left(f;x\right)={\left(1+q\right)}^{-n}\overset{n}{\underset{k=0}{\sum }}\left(\begin{array}{c} n\\ k \end{array}\right)q^{n-k}{\overset{\sim }{S}}_{k}, \end{array}$$

of the conjugate series (2) is given by

(19) $$\begin{array}{c} {||{\overset{\sim }{\sigma }}_{n}(f;x)-\overset{\sim }{f}(x)||}_{p}=O\left(\xi \left(\frac{1}{\sqrt{n}}\right){\left(n\right)}^{1/2p}\right). \end{array}$$

In order to prove our theorem, we need the following lemma.

**Lemma 2**

If  0 < *t* ≤ *π*, then

(20) $$\begin{array}{c} {\left(1+q\right)}^{-n}{\left(1+q^{2}+2q\cos t\right)}^{n/2}\leq e^{-2qt^{2}n/{\left\{\pi (1+q)\right\}}^{2}}. \end{array}$$

**Proof**

We have

(21) (1+q)−2(1+q2+2qcos⁡t) =1−4qsin2(t/2)(1+q)2 ≤1−4qt2  π2(1+q)2 ≤e−4qt2/{π(1+q)}2,

since  *e* ^*x*^(1 − *x*) < 1  when  0 < *x* < 1. Therefore,

(22) $$\begin{array}{c} {\left(1+q\right)}^{-n}{\left(1+q^{2}+2q\cos t\right)}^{n/2}\leq e^{-2qt^{2}n/{\left\{\pi (1+q)\right\}}^{2}}. \end{array}$$

**Proof of Theorem 1**

The  *k*th  partial sum of the conjugate series of the Fourier series (2) is given by

(23) S~k(f;x)=−12π∫0πcot(t2)ψ(t)dt +12π∫0πcos⁡(k+1/2)tsin(t/2)ψ(t)dtS~k(f;x)−(−12π∫0πcot⁡(t2)ψ(t)dt)  =12π∫0πcos⁡(k+1/2)tsin⁡(t/2)ψ(t)dt.

Taking Euler (*E*, *q*) means, we get

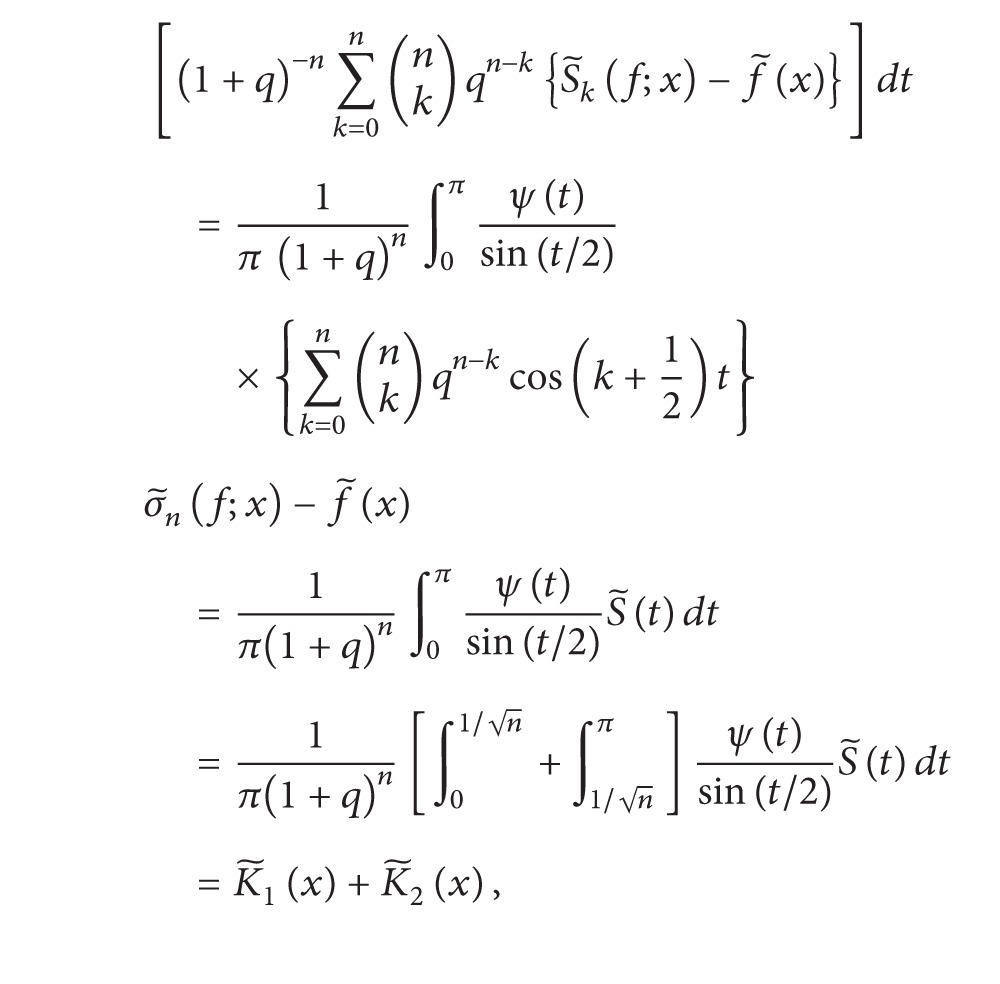
(24)

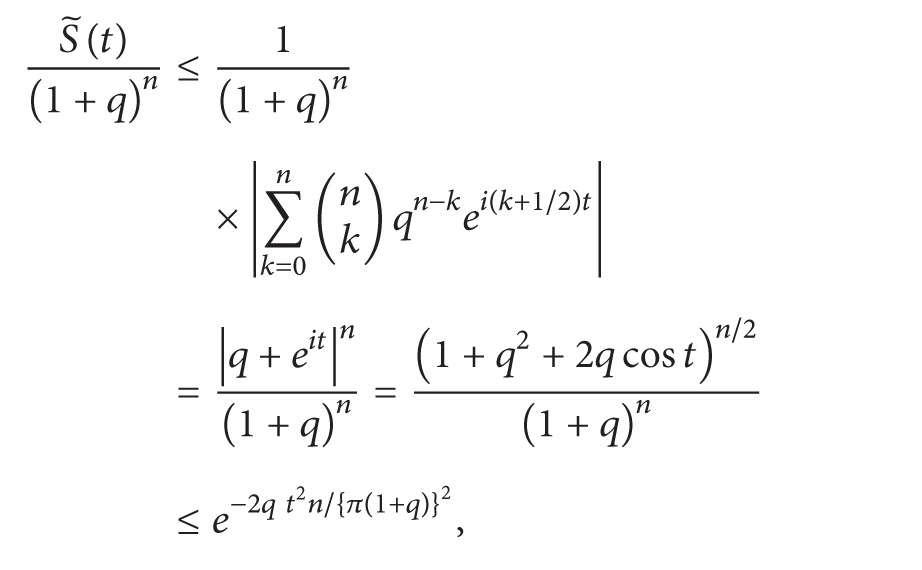
(25)
using Lemma 2.

Clearly,

(26) |ψ(x+t)−ψ(x)|≤|f(u+x+t)−f(u+x)| +|f(u+x)−f(u−x−t)|.

Hence, by Minkowski's inequality,

(27) {∫02π|(ψ(x+t)−ψ(x))|pdx}1/p ≤{∫02π|(f(u+x+t)−f(u+x))|pdx}1/p  +{∫02π|f(u+x)−  (f(u−x−t))|pdx}1/p =O(ξ(t)).

Then,  *f* ∈ Lip (*ξ*(*t*), *p*)⇒*ψ* ∈ Lip (*ξ*(*t*), *p*).

Using Hölder's inequality,  *ψ*(*t*) ∈ Lip (*ξ*(*t*), *p*), condition (16),  sin*t* ≥ (2*t*/*π*), lemma, and second mean value theorem for integrals, we have

(28) |K~1(x)|=O{∫01/n(|ψ(t)|)pdt}1/p ×{∫01/n((1+q)−nS~(t)sin⁡(t/2))p′dt}1/p′,

where

(29) p′=pp−1|K~1(x)|=O{∫01/n(|ξ(t)t1/p|)pdt}1/p ×{∫01/n(e−2qt2n/{π(1+q)}2|sin⁡(t/2)|  )p′dt}1/p′=O(ξ(1n)){∫01/nt−p′dt}1/p′=O(ξ(1n)(n)1/2p).

Now,

(30) |K~2(x)|=O[∫1/nπ|ψ(t)|sin(t/2)(1+q)−n(1+q2+2qcos⁡t)n/2|R(t)|dt]=O{∫1/nπ|ψ(t)|sin(t/2)(1+q)−n   ×(1+q2+2qcos⁡t)n/2dt}=O{∫1/nπ|ψ(t)|sin(t/2)e−2qt2n/{π(1+q)}2dt}=O[1n∫1/nπψ(t)sin(t/2)   ×{∂∂t(e−2qt2n/{π(1+q)}2)}dt].

Using Hölder's inequality,  *ψ*(*t*) ∈ Lip (*ξ*(*t*), *p*), and condition (17), we have

(31) |K~2(x)|=O[1n∫1/nπ(ξ(t)t1/p+2)pdt]1/p ×[∫1/nπ{∂∂t(e−2qt2n/{π(1+q)}2)}p′dt]1/p′=O(ξ(1n)(n)1/2p).

Combining (24) with (31), we have

(32) $$\begin{array}{c} {||{\overset{\sim }{\sigma }}_{n}(f;x)-\overset{\sim }{f}(x)||}_{p}=O\left(\xi \left(\frac{1}{\sqrt{n}}\right){\left(n\right)}^{1/2p}\right), \end{array}$$

which completes the proof of the theorem.

## 3. Corollaries

The following corollaries may be derived from our theorem.

**Corollary 3**

If  *ξ*(*t*) = *t* ^*α*^, then the degree of approximation of a function$\;\overset{\sim }{f}\left(x\right)$, conjugate of  *f* ∈ Lip (*α*, *p*),  1/*p* < *α* < 1, by Euler's means  (*E*, *q*)  of the conjugate series of the Fourier series (2) is given by

(33) $$\begin{array}{c} {||{\overset{\sim }{\sigma }}_{n}\left(f;x\right)-\overset{\sim }{f}\left(x\right)||}_{p}=O\left(\frac{1}{n^{(\alpha p-1)/2p}}\right). \end{array}$$

**Corollary 4**

If  *p* → *∞*  in Corollary 3, then, for  0 < *α* < 1,

(34) $$\begin{array}{c} {||{\overset{\sim }{\sigma }}_{n}\left(f;x\right)-\overset{\sim }{f}\left(x\right)||}_{\infty }=O\left(\frac{1}{n^{\alpha /2}}\right). \end{array}$$
